# *rac*-2,3-Dibromo­propionamide

**DOI:** 10.1107/S160053681205132X

**Published:** 2013-01-04

**Authors:** Robert Köppen, Franziska Emmerling, Matthias Koch

**Affiliations:** a BAM Federal Institute for Materials Research and Testing, Department of Analytical Chemistry, Reference Materials, Richard-Willstätter-Strasse 11, D-12489 Berlin-Adlershof, Germany

## Abstract

The racemic title compound, C_3_H_5_Br_2_NO, was crystallized from methanol. In the crystal, adjacent mol­ecules are linked through N—H⋯O hydrogen bonds, forming chains along the *c-*axis direction. These chains are linked through N—H⋯O hydrogen bonds, forming an undulating two-dimensional network lying parallel to the *bc* plane. There are also short Br⋯Br contacts present [3.514 (3) Å].

## Related literature

For the crystal structure of the starting material, see: Zhou *et al.* (2007 ▶). For the development and application of acryl­amide analysis in food, see: Rosén & Hellenäs (2002 ▶); Hashimoto (1976 ▶); Nemoto *et al.* (2002 ▶); Cheng *et al.* (2006 ▶); Mizukami *et al.* (2006 ▶), Zhang *et al.* (2005 ▶, 2006 ▶). For halogen inter­actions, see: Pedireddim *et al.* (1994 ▶).
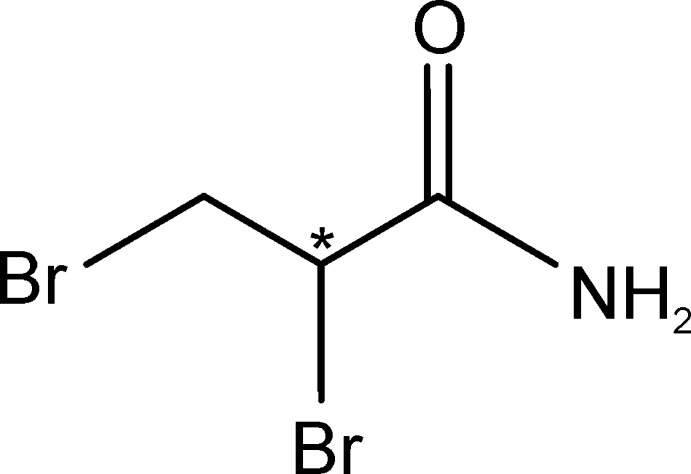


## Experimental

### 

#### Crystal data

C_3_H_5_Br_2_NO
*M* *_r_* = 230.88
Monoclinic, 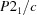

*a* = 11.926 (3) Å
*b* = 6.5911 (14) Å
*c* = 8.991 (2) Å
β = 103.574 (14)°
*V* = 687.0 (3) Å^3^
*Z* = 4
Mo *K*α radiation
μ = 11.70 mm^−1^
*T* = 296 K
0.14 × 0.11 × 0.05 mm

#### Data collection

Bruker APEX CCD area-detector diffractometer
Absorption correction: multi-scan (*SADABS*; Bruker, 2001 ▶) *T*_min_ = 0.23, *T*_max_ = 0.56
4500 measured reflections
1556 independent reflections
470 reflections with *I* > 2σ(*I*)
*R*_int_ = 0.181

#### Refinement

*R*[*F* ^2^ > 2σ(*F* ^2^)] = 0.066
*wR*(*F* ^2^) = 0.184
*S* = 0.77
1556 reflections
64 parameters
H-atom parameters constrained
Δρ_max_ = 0.86 e Å^−3^
Δρ_min_ = −0.50 e Å^−3^

### 

Data collection: *SMART* (Bruker, 2001 ▶); cell refinement: *SAINT* (Bruker, 2001 ▶); data reduction: *SAINT*; program(s) used to solve structure: *SHELXS97* (Sheldrick, 2008 ▶); program(s) used to refine structure: *SHELXL97* (Sheldrick, 2008 ▶); molecular graphics: *SHELXTL* (Sheldrick, 2008 ▶) and *ORTEPIII* (Burnett & Johnson, 1996 ▶); software used to prepare material for publication: *SHELXTL*.

## Supplementary Material

Crystal structure: contains datablock(s) I, global. DOI: 10.1107/S160053681205132X/bg2488sup1.cif

Supplementary material file. DOI: 10.1107/S160053681205132X/bg2488Isup2.mol

Structure factors: contains datablock(s) I. DOI: 10.1107/S160053681205132X/bg2488Isup3.hkl

Supplementary material file. DOI: 10.1107/S160053681205132X/bg2488Isup4.cml

Additional supplementary materials:  crystallographic information; 3D view; checkCIF report

## Figures and Tables

**Table 1:** Hydrogen-bond geometry (Å, °)

*D*—H⋯*A*	*D*—H	H⋯*A*	*D*⋯*A*	*D*—H⋯*A*
N1—H1⋯O1^i^	0.86	2.55	3.185 (11)	132
N1—H2⋯O1^ii^	0.86	2.09	2.942 (12)	173

Symmetry codes: (i) 
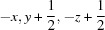
; (ii) 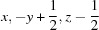.

**Fig. 1.**
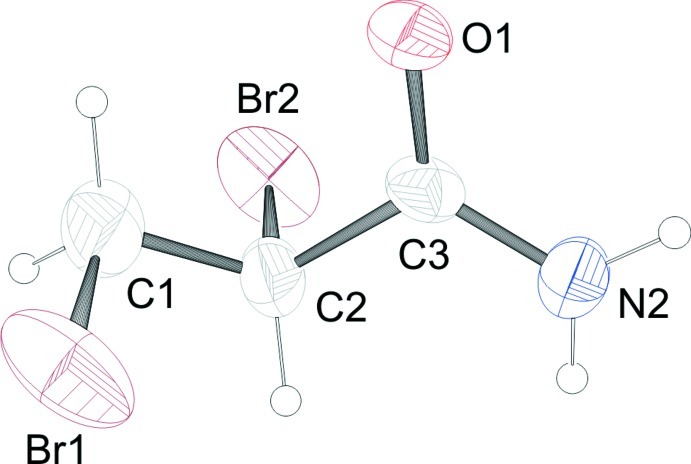
: ORTEP representation of the title compound with atomic labeling shown with 30% probability displacement ellipsoids.

**Fig. 2.**
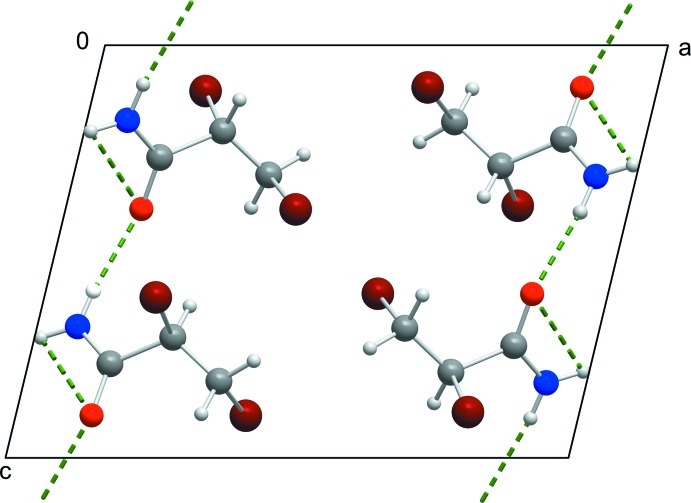
: View of the unit cell of the title compound along [010] showing the hydrogen-bonded chains along the [001] direction. Hydrogen bonds are drawn as dashed green lines.

## supplementary crystallographic information

### Comment

Since April 2002 researchers from the Swedish National Food Administration and Stockholm University reported the detection of acrylamide (AA) in fried and baked foods (Rosén & Hellenäs, 2002) for the first time, a lot of attention was attracted to studies and investigations of AA in a wide variety of food matrices. As a result, the number of published papers concerning the development and application of AA analysis in food has increased enormously in the past years and led to an extensive bibliography.

The discovery of AA in food was, and still is, a matter of public concern, due to its neurotoxic, clastogenic and probably carcinogenic effects. For the determination of AA various sample handling techniques such as defatting, liquid–liquid extraction, solid-phase extraction using different types of cartridges were applied followed either by high-performance liquid chromatography (HPLC) with mass spectrometric (MS) or diode array detection (DAD) or by gas chromatography (GC) with electron-capture (ECD) or MS detection.

When using GC—MS, AA can be analysed without derivatization but is normally brominated to form a derivative revealing improved GC properties (more volatile and less polar). A conversion of AA to 2,3-dibromopropionamide (2,3-DBPA) is usually performed by addition of anhydrous potassium bromide, hydrobromic acid and a saturated solution of bromine in water (protocol by Hashimoto, 1976) or by using KBr-KBrO_3_ to avoid elemental bromine (Nemoto *et al.*, 2002). The resulting 2,3-DBPA is extracted from aqueous solutions and can be more easily detected with GC-ECD/MS. However, different studies have shown that under certain conditions, 2,3-DBPA can be decomposed to the more stable derivative 2-bromopropenamide (2-BPA) during GC-analysis. Therefore, triethylamine is meanwhile used to convert 2,3-DBPA to the stable 2-BPA in a second derivatization step prior to GC analysis. The compound crystallizes in the monoclinic space group *P*2_1_/c. The molecular structure of the compound and the atom-labeling scheme are displayed in Fig 1. Within each molecule an intramolecular N—H···O hydrogen bond between the amide and the carboxyl group is formed. Adjacent molecules are connected *via* N—H···O hydrogen bonds to form chains along the [0 0 1] direction (see dashed bonds bonds in Fig. 2). Between two of the bromine atoms a type I halogene interactions can be observed (Pedireddim *et al.*, 1994). These halogen···halogen contacts C—*X*···*X*—C are defined as type I if the C—*X*···*X* angle α1 is equal or nearly equal to the *X*···*X*—C angle α2. Type I contacts arise as a result of close packing about an inversion center.

### Experimental

A 250 mL three-necked round-bottomed flask fitted with a thermometer, a magnetic stirrer, a condenser and a 100 mL dropping funnel, was charged with 60 mL chloroform followed by 5 g (70.33 mmol) of acrylamide. The solution was cooled to 0–5°C in an ice bath, and bromine (11.24 g, 70.33 mmol) dissolved in 20 mL chloroform was added cautiously (dropwise) over a period of about 4 h under vigorous stirring. After the addition, stirring in the cold was continued for 1 h followed by stirring at room temperature for 2 h. Evaporation of the chloroform (rotary evaporator) and subsequent recrystallization (methanol) of the residue affords the product in about 94.3% yield (mp 132.8°C/1.013 bar). Despite repeated recrystallization it was not possible to completely avoid builtups degradation products. Therfore, larger crystals were chosen for X-ray single-crystal structure analysis to reduce the influence of buitups on the crystal surface.

### Refinement

Decomposition of the crystals during the measurments was observed, but repeated measurements using different crystals did not lead to a better dataset. All H-atoms were positioned geometrically and refined using a riding model with d(C—H) = 0.93 Å, *U*_iso_=1.2*U*_eq_ (C) for aromatic 0.98 Å, *U*_iso_ = 1.2*U*_eq_ (C) for CH, 0.97 Å, *U*_iso_ = 1.2*U*_eq_ (C) for CH_2_, 0.96 Å, and 0.82 Å, *U*_iso_ = 1.5*U*_eq_ (N) for the amino group.

### Crystal data

**Table:**

C_3_H_5_Br_2_NO	*F*(000) = 432
*M**_r_* = 230.88	*D*_x_ = 2.232 Mg m^−^^3^
Monoclinic, *P*2_1_/*c*	Mo *K*α radiation, λ = 0.71073 Å
Hall symbol: -P 2ybc	Cell parameters from 756 reflections
*a* = 11.926 (3) Å	θ = 2.5–20.6°
*b* = 6.5911 (14) Å	µ = 11.70 mm^−^^1^
*c* = 8.991 (2) Å	*T* = 296 K
β = 103.574 (14)°	Block, colourless
*V* = 687.0 (3) Å^3^	0.14 × 0.11 × 0.05 mm
*Z* = 4	

### Data collection

**Table:**

Bruker APEX CCD area-detector diffractometer	1556 independent reflections
Radiation source: fine-focus sealed tube	470 reflections with *I* > 2σ(*I*)
Graphite monochromator	*R*_int_ = 0.181
ω/2θ scans	θ_max_ = 27.5°, θ_min_ = 1.8°
Absorption correction: multi-scan (*SADABS*; Bruker, 2001)	*h* = −15→15
*T*_min_ = 0.23, *T*_max_ = 0.56	*k* = −8→8
4500 measured reflections	*l* = −10→11

### Refinement

**Table:**

Refinement on *F*^2^	Primary atom site location: structure-invariant direct methods
Least-squares matrix: full	Secondary atom site location: difference Fourier map
*R*[*F*^2^ > 2σ(*F*^2^)] = 0.066	Hydrogen site location: inferred from neighbouring sites
*wR*(*F*^2^) = 0.184	H-atom parameters constrained
*S* = 0.77	*w* = 1/[σ^2^(*F*_o_^2^) + (0.0796*P*)^2^] where *P* = (*F*_o_^2^ + 2*F*_c_^2^)/3
1556 reflections	(Δ/σ)_max_ < 0.001
64 parameters	Δρ_max_ = 0.86 e Å^−^^3^
0 restraints	Δρ_min_ = −0.50 e Å^−^^3^

### Special details

**Table:**

Geometry. All e.s.d.'s (except the e.s.d. in the dihedral angle between two l.s. planes) are estimated using the full covariance matrix. The cell e.s.d.'s are taken into account individually in the estimation of e.s.d.'s in distances, angles and torsion angles; correlations between e.s.d.'s in cell parameters are only used when they are defined by crystal symmetry. An approximate (isotropic) treatment of cell e.s.d.'s is used for estimating e.s.d.'s involving l.s. planes.
Refinement. Refinement of *F*^2^ against ALL reflections. The weighted *R*-factor *wR* and goodness of fit *S* are based on *F*^2^, conventional *R*-factors *R* are based on *F*, with *F* set to zero for negative *F*^2^. The threshold expression of *F*^2^ > σ(*F*^2^) is used only for calculating *R*-factors(gt) *etc*. and is not relevant to the choice of reflections for refinement. *R*-factors based on *F*^2^ are statistically about twice as large as those based on *F*, and *R*- factors based on ALL data will be even larger.

### Fractional atomic coordinates and isotropic or equivalent isotropic displacement parameters (Å^2^)

**Table:**

	*x*	*y*	*z*	*U*_iso_*/*U*_eq_	
Br1	0.40891 (12)	0.3123 (3)	0.39780 (17)	0.1139 (8)	
Br2	0.19816 (12)	−0.1953 (2)	0.11104 (17)	0.1065 (7)	
O1	0.1347 (5)	0.0988 (12)	0.3970 (9)	0.067 (2)	
N1	0.0718 (7)	0.2806 (13)	0.1819 (10)	0.072 (3)	
H1	0.0110	0.3215	0.2088	0.087*	
H2	0.0844	0.3184	0.0957	0.087*	
C1	0.3463 (10)	0.030 (2)	0.3142 (14)	0.107 (5)	
H4	0.3995	−0.0396	0.2652	0.129*	
H5	0.3291	−0.0550	0.3939	0.129*	
C2	0.2455 (8)	0.0907 (19)	0.2077 (12)	0.077 (3)	
H3	0.2622	0.1865	0.1325	0.092*	
C3	0.1452 (8)	0.1613 (15)	0.2720 (13)	0.054 (3)	

### Atomic displacement parameters (Å^2^)

**Table:**

	*U*^11^	*U*^22^	*U*^33^	*U*^12^	*U*^13^	*U*^23^
Br1	0.1008 (11)	0.1465 (16)	0.1073 (13)	−0.0630 (10)	0.0505 (9)	−0.0552 (10)
Br2	0.1118 (12)	0.0981 (12)	0.1182 (13)	−0.0294 (8)	0.0443 (9)	−0.0549 (9)
O1	0.070 (5)	0.080 (5)	0.059 (5)	0.007 (4)	0.034 (4)	0.011 (4)
N1	0.067 (6)	0.082 (7)	0.071 (6)	0.025 (5)	0.022 (5)	0.021 (5)
C1	0.085 (9)	0.163 (15)	0.088 (10)	0.048 (9)	0.048 (8)	0.029 (9)
C2	0.050 (6)	0.115 (10)	0.069 (8)	0.019 (6)	0.021 (6)	0.023 (7)
C3	0.063 (7)	0.057 (8)	0.050 (7)	0.001 (5)	0.031 (6)	−0.003 (6)

### Geometric parameters (Å, º)

**Table:**

Br1—C1	2.077 (15)	C1—C2	1.407 (14)
Br2—C2	2.097 (12)	C1—H4	0.9700
O1—C3	1.231 (10)	C1—H5	0.9700
N1—C3	1.307 (12)	C2—C3	1.518 (13)
N1—H1	0.8597	C2—H3	0.9800
N1—H2	0.8604		
			
C3—N1—H1	120.1	C1—C2—C3	116.9 (10)
C3—N1—H2	119.9	C1—C2—Br2	97.5 (9)
H1—N1—H2	120.0	C3—C2—Br2	105.9 (7)
C2—C1—Br1	99.8 (9)	C1—C2—H3	111.8
C2—C1—H4	111.8	C3—C2—H3	111.8
Br1—C1—H4	111.8	Br2—C2—H3	111.8
C2—C1—H5	111.8	O1—C3—N1	124.8 (9)
Br1—C1—H5	111.8	O1—C3—C2	120.2 (10)
H4—C1—H5	109.5	N1—C3—C2	114.9 (10)
			
Br1—C1—C2—C3	−74.1 (11)	Br2—C2—C3—O1	80.6 (10)
Br1—C1—C2—Br2	173.7 (4)	C1—C2—C3—N1	156.6 (12)
C1—C2—C3—O1	−26.7 (17)	Br2—C2—C3—N1	−96.1 (9)

### Hydrogen-bond geometry (Å, º)

**Table:**

*D*—H···*A*	*D*—H	H···*A*	*D*···*A*	*D*—H···*A*
N1—H1···O1^i^	0.86	2.55	3.185 (11)	132
N1—H2···O1^ii^	0.86	2.09	2.942 (12)	173

Symmetry codes: (i) −*x*, *y*+1/2, −*z*+1/2; (ii) *x*, −*y*+1/2, *z*−1/2.
